# Topical effect of a specific spot-on treatment made of natural ingredients in rabbits (*Oryctolagus cuniculus*) with skin problems: A pilot study

**DOI:** 10.14202/vetworld.2020.1760-1763

**Published:** 2020-09-03

**Authors:** Galia Sheinberg Waisburd, Alberto Martin Cordero, Camilo Romero Núñez, Laura Miranda Contreras, Rafael Heredia Cárdenas, Linda G. Bautista Gómez

**Affiliations:** 1 Veterinary Center Mexico, Cincinnati Street #22, Benito Juárez, Ciudad de los Deportes, 03710 Mexico city, Mexico; 2 VETDERM: Specialized Veterinary Dermatology, Guadalajara, Jalisco, Mexico; 3 Dermavet Veterinary Hospital, José de la Luz Blanco, Mz. 187, Lt. 33, Col. Santa Martha Acatitla, Mexico city, Mexico; 4 UAEM Amecameca University Center, Autonomous Mexico State University, Km. 2.5 Amecameca-Ayapango Highway, 56900 Amecameca de Juárez, Mexico

**Keywords:** essential oils, polyunsaturated fatty acids, rabbits, skin, Vitamin E

## Abstract

**Background and Aim:**

Rabbits often experience skin diseases. The beneficial effects of plant extracts and essential oils are well known in other species, but the properties of these natural ingredients have not been evaluated in rabbits *in vivo*. The objective of the current study was to evaluate the effect of a topical, commercial solution made of essential oils, plant-extracted polyunsaturated fatty acids, and Vitamin E on rabbits suffering from skin problems.

**Materials and Methods:**

Thirty New Zealand rabbits (no sex distinction) were included in this study, with an average weight of 2–3 kg. The rabbits were divided into two groups: The first group was treated with a topical solution made from natural ingredients, and the second was a control group. The rabbits’ hair and skin conditions were evaluated on days 1, 14, 28, and 35 after treatment. Data were analyzed using a Kruskal–Wallis range test.

**Results:**

Significant differences were determined in terms of glossy hair variability on days 28 and 35 (p≤0.0001). On days 14-35, hair loss was determined to have reduced (p=0.001), and flaking and odor improved in the treatment group, showing increased scores and significant differences (p=0.0001). By contrast, the control group showed stable overall skin and hair score and an increase in the dryness score.

**Conclusion:**

The topical application of essential oils and polyunsaturated fatty acids with Vitamin E was able to improve hair shine and skin hydration and reduce flaking, bad odor, and hair loss, improving the general, and cutaneous aspect of rabbits.

## Introduction

For centuries, rabbits (*Oryctolagus cuniculus*) have been part of the daily lives of humans [1]. At present, they are popular as companion animals. They are valued pets, identified to have become the small animal species with the third most visits to veterinary practices [2]. In veterinary practice, skin diseases represent a significant problem. The general approach to skin diseases in rabbits is similar to that for dogs and cats, which includes obtaining a complete history, feeding, and housing information, performing a clinical examination, and performing complementary tests [3]. Most of the skin conditions can be attributed to a wide variety of pathogens (parasites, fungi, bacteria, and viruses). Furthermore, non-infectious diseases in domestic rabbits are increasingly being reported, possibly because owners are searching for better and more complete care for their rabbits [4].

New natural substances are continually being isolated and identified for treatment, for example, the antibacterial, anthelmintic, and antifungal effects of plant extracts and essential oils. In general, their properties and activities have been examined mainly under *in vitro* conditions. However, to improve their impact, *in vivo* experiments are also required [5]. Fatty acids have already been used in treating humans, dogs, and cats with atopic dermatitis [6]. The topical administration or supplementation of the diet with free-fatty acids could stimulate the production of endogenous lipids, which, in turn, can contribute to the formation and improvement of the epidermal barrier [7] and decrease the production of inflammatory mediators and the inhibition of cellular immunity [6]. Although they are known to be less effective than glucocorticoids and cyclosporine, fatty acids are deemed safer adjunctive therapy compared to other anti-inflammatory therapies [8].

Unsaturated fatty acids and essential oils have also been proven to improve the skin layer’s odor and gloss and reduce scale formation [6]. In dogs, cutaneous malodor has been associated with the increase of certain bacterial genres present in the skin. Skin malodor does not directly induce morbidity or mortality, but it can affect the quality of human-animal bond, because unsuccessful control of pet odor can become frustrating and require frequent bathing and dietary changes [9]. The use of natural substances, such as fatty acids, in the breeding of rabbits, is a promising path to improve aspects of their health and well-being [5]. Essential oils have also shown efficacy against pathogens in otitis externa, such as *Malassezia* in dogs [10]. Thus, this study aims to evaluate the effect of a commercial topical solution composed of essential oils, essential fatty acids from plants, and Vitamin E on the clinical signs of skin disorders in rabbits.

## Materials and Methods

### Ethical approval

The Ethics Committee of the UAEM Amecameca University Center approved the study protocol [Approval no. (CBE/CUA/2017/51)].

### Study area and animals

This study was conducted in the UAEM Amecameca University Center, Mexico, from November to December 2018. All animals and procedures were performed in compliance with the practices for the care of experimental animals and recommendations according to the Mexican norm (NOM-062-ZOO-1999). We included 30 rabbits of an indistinct genus, New Zealand breed, with an average weight of 2-3 kg and with cutaneous disorders. The rabbits were provided a diet based on commercial rabbit feed and water *ad libitum*. The rabbits did not receive any pharmacological or systemic treatment previous to the study. The inclusion criteria were as follows: Rabbits with dry skin, loss of gloss, opaque or brittle coat, excessive hair loss, and bad odor. All rabbits underwent individual physical evaluation. The animals were registered in files with their corresponding data.

### Experimental design

Thirty rabbits were randomly divided into two groups. Fifteen were treated with the topical solution containing essential oils (clove, camphor, wintergreen, rosemary, turmeric, oregano, lavender, peppermint, tea tree, and cedar), essential fatty acids (hemp seed and neem seed oils), and Vitamin E. The spot-on solution was applied directly to the skin by separating the hair from the interscapular region, at a dosage according to the animal’s weight. Rabbits weighing up to 5 kg were treated with 0.06 ml of solution/kg. The solution was applied once per week for 4 weeks. Meanwhile, the remaining 15 rabbits formed the control group. All 30 rabbits were evaluated on days 1, 14, 28, and 35 using a clinical score for areas such as the face, head, neck, sternum, chest, groin, abdomen, back, sides, hind limbs, posterior end, perianal, perigenital, and tail. A description of the fur and skin conditions of each animal was made using the following criteria and scores: Very dull (1) to very shiny (7) for coat shine, very severe (1) to absent (7) for hair loss and flaking, and very bad (1) to very pleasant (7) for body odor. Skin and hair balance were also scored as very greasy (1), greasy (2), slightly greasy (3), and normal (4) and very dry (1), dry (2), slightly dry (3), and normal (4), respectively. The evaluations of the rabbit’s skin were performed by the same researcher for each rabbit. This protocol was applied according to the manufacturer’s recommendations.

### Statistical analysis

The Shapiro–Wilk normality test was applied to determine the distribution of the data. The data did not present a normal distribution. The data were analyzed using the Kruskal–Wallis test to compare the variables between the two groups, with an alpha of 0.05 set for statistical significance.

## Results

All rabbits concluded the study. Skin evaluations were performed on all rabbits every 2 weeks and again on day 35 (1 week after the end of treatment). All 30 rabbits presented dermatological disorders with no difference in any of the evaluation criteria on day 1. For the coat shine criteria, no significant differences were determined between groups on day 14. However, there were significant differences between days 28 and 35. The scores of the treated group increased at each time point, whereas little variation was observed in the control group scores. The scores for hair loss, flaking, and odor all improved, with significant differences in the treated group at all-time points on days 14, 28, and 35.

Conversely, the control group scores have decreased for all criteria, showing significant differences in hair loss, flaking, and odor between groups at all-time points on days 14, 28, and 35. For skin balance, between greasiness and dryness, scores improved in the treated group and stabilized, indicating a normalized skin and hair condition (4). The scores for the control group, however, deteriorated, indicating drier or greasy skin and hair, with significant differences on days 28 and 35. The scores for the treatment group increased for each evaluated criterion, whereas the control group scores decreased (Table-1).

**Table-1:** Mean comparison of skin and coat condition in rabbits with dermatological problems treated with the spot on and control.

Fur and skin conditions	Day 1	Day 14	Day 28	Day 35
Coat shine				
Spot-on	2.00	2.46	4.40	5.00
Control	2.13	2.13	2.20	2.06
Chi-square	0.25	1.17	23.49	23.89
p-value	0.61	0.27	0.0001	0.0001
Hair loss				
Spot on	4.53	4.86	6.46	6.86
Control	4.26	3.00	2.46	2.26
Chi-square	0.08	9.89	23.10	24.53
p-value	0.76	0.001	0.0001	0.0001
Scales				
Spot on	4.93	5.80	6.40	6.66
Control	4.53	2.26	2.06	2.00
Chi-square	1.07	22.78	23.23	23.94
p-value	0.30	0.0001	0.0001	0.0001
Odor				
Spot on	3.53	5.13	5.66	5.86
Control	4.00	3.13	2.46	2.13
Chi-square	1.27	17.59	23.47	25.99
p-value	0.25	0.0001	0.0001	0.0001
Greasy/dry hair/skin				
Spot on	3.26	3.73	3.93	4.00
Control	3.13	3.06	2.40	2.20
Chi-square	0.28	7.26	23.99	26.85
p-value	0.59	0.007	0.0001	0.0001

Kruskal–Wallis test, p*≤*0.05

## Discussion

In rabbits, skin diseases are identified as one of the most common reasons for veterinary consultations. Oils and plant extracts have been administered orally for a wide variety of purposes since they were reported to possess antimicrobial activities and even anticoccidial effects in rabbits [11]. To the best of our knowledge, this is the first report on the use of a topical spot-on solution made of essential oils, essential fatty acids, and Vitamin E for the resolution of cutaneous disorders in rabbits.

Essential oils have been reported to offer various benefits because of its analgesic, antiseptic, antimicrobial, antifungal, carminative, diuretic, spasmolytic, hyperemic, and stimulant properties [12]. *In vitro*, they have been shown to have a high antimicrobial effect against a wide range of dermatological pathogens [13]. Transepidermal water loss (TEWL) has been measured in normal dogs and dogs with canine atopic dermatitis, as well as the level of pruritus before and after the application of a topical formulation with essential oils and unsaturated fatty acids. This resulted in a decrease in pruritus and a significant difference between the TEWL values of healthy and atopic dogs on the abdomen (p = 0.0181) and back (p = 0.0123) [6]. A study conducted by Blaskovic *et al*. [8] evaluated the effect of polyunsaturated fatty acids and essential oils applied topically to 48 dogs with canine atopic dermatitis and demonstrated an improvement in clinical signs (pruritus and lesions) after 8 weekly topical treatments. Dietary supplementation with essential fatty acids in dogs with canine atopic dermatitis has been shown to reduce pruritus and other clinical signs, leading to a statistically significant reduction (p < 0.0001) from day 0 to day 84, in addition to a sparing effect on the amount of steroid medication needed to control this condition [14].

Bensignor and Bordeau [15], in two studies conducted with domestic carnivores, have shown that the components of spot-on treatments (unsaturated fatty acids and essential oils) maintain or restore the hydrolipidic film and increase epidermal hydration. This is because they reduce TEWL, as well as improve the animals’ appearance, according to the same evaluation criteria used in the present study (coat gloss, scaling, odor, skin appearance, and hair loss). This efficacy is consistent with the results of the current study, in which all rabbits initially presented some skin disorders. From day 14 to day 35, hair loss, scaling, and odor reportedly decreased in the treated group, resulting in a significant difference for each criterion (p ≤ 0.0001). The glossy hair variable was also determined to be significantly different from days 28 to 35. As reported in a model of canine skin, essential oils and the formulation of polyunsaturated fatty acids resulted in a thicker epidermis with a higher number of viable cell layers, a more dense and compact stratum corneum, and a more continuous basal membrane, in addition to a significant increase in ceramide content, which improves the condition of the skin [7]. Meason–Smith *et al*. [9] showed that an increase of certain bacterial genera (*Psychrobacter* and *Pseudomonas*) in the skin of bloodhounds was associated with cutaneous odor and that the use of a topical product based on essential oils and essential fatty acids derived from plants, similar to that used in the present study, significantly improved cutaneous odor and modified the structure of the bacterial community in the treatment group after four topical applications once per week.

Because of its role as a barrier, the skin is often exposed to an oxidative environment, which includes air pollutants, ultraviolet radiation, released oxidants (from normal metabolism), parasites, and aerobic microorganisms [16]. Vitamin E, an antioxidant concentrated in cell membranes, is an important component of sebum, secreted continuously on the surface of the skin [17]. This topically applied vitamin increases peripheral blood flow in local skin, leading to accelerated hair growth in rabbits. Its effects have been observed as early as 2 weeks after its application, and the full effect becomes evident after 4 weeks [18]. Several studies suggest that Vitamin E contains anticancer, photoprotective, and stabilizing properties of the cutaneous barrier when administered both topically and orally [19]. These results coincide with this study, in which the spot-on treatment was applied once a week for 4 weeks. Hair loss was reduced after the 2^nd^ week of treatment.

One factor that should be considered when practicing medicine in rabbits is handling, as rabbits can be easily stressed, including bathing [20]. The application of topical products favors stress-free management and is deemed easy to administer. In addition, topical therapy reaches diseased tissue directly, which allows for higher concentrations of the drug at the affected site [21], including essential oils, which consist mainly of lipophilic, small, and non-polar molecules that can easily penetrate the skin and affect the site [12].

## Conclusion

Our findings showed that the topical cutaneous administration of essential oils, fatty acids, and Vitamin E applied weekly as a spot-on treatment exhibited clinical improvement of the cutaneous aspect in rabbits. As no adverse reactions were observed, this was identified to be a safe and effective treatment.

## Authors’ Contributions

GSW and AMC: Participated in writing, interpreting, and editing the manuscript. CRN: Substantial contribution to the concept and design of the study; contribution to data collection, interpretation, and contribution to manuscript preparation and critical revision. LMC: Participated in writing, interpreting, and editing the manuscript and critical revision. RHC: Substantial contribution to the statistical data analysis and interpretation and critical revision. LGBG: Contribution to data collection and critical revision. All authors read and approved the final manuscript.
